# Posttraumatic Immune Response and Its Modulation

**DOI:** 10.1155/2012/731563

**Published:** 2012-03-27

**Authors:** Frank Hildebrand, Sascha Flohe, Loek Leenen, Martijn van Griensven, Michael Frink

**Affiliations:** ^1^Trauma Department, Hannover Medical School, 30625 Hannover, Germany; ^2^Department of Trauma and Hand Surgery, Düsseldorf University Hospital, 40204 Düsseldorf, Germany; ^3^Department of Trauma Surgery, UMC Utrecht AZU Location, 3508 GA Utrecht, The Netherlands; ^4^Trauma Department, University Hospital on The Right of The River Isar, 81675 Munich, Germany

Following accidental trauma, an inflammatory response is induced involving hormonal, metabolic, and immunological mediators. This posttraumatic immune response is a physiological process required for tissue repair and regulation of the healing process. An overwhelming response after major trauma causes a disbalance between pro- and anti-inflammatory mediators resulting in complications like an increased susceptibility to infection, sepsis, and the Multiorgan-Dysfunction-Syndrome (MODS) [1]. Therefore, profound knowledge of this inflammatory response is a major interest in order to anticipate and control these potential posttraumatic complications.

Following major trauma, a local release of mediators such as cytokines, acid metabolites, and histamine increases the capillary permeability resulting in tissue edema and local infiltration of immunocompetent cells. Intrinsic leukocytes and affected endothelial cells produce and release pro- and anti-inflammatory cytokines acting locally as well as on remote cells in different organs like the lung [1, 2]. In this issue P. Mommsen et al. investigated the role of IL-6 on cytokine production capacity of splenocytes after femoral shaft fracture, isolated or in combination with hemorrhage in male mice. The authors showed that an isolated femoral fracture resulted in a suppression of *in vitro* cytokine synthesis of splenocytes, which was further enhanced in case of an additional hemorrhagic shock. In IL-6 knockout mice, the splenic immunodepression after femoral fracture and hemorrhagic shock attenuated. These results give evidence that the posttraumatic modulation of IL-6 synthesis might be a potential target for therapeutic interventions. As contradictory findings in animal models of trauma and sepsis impede a rationale for therapies directed against IL-6 in general, selective inhibition of IL-6 transsignaling using soluble IL-6R seems to be a potential treatment strategy [3].

P. Kobbe et al. (in this issue) described the effects of inhalative IL-10 administration on the pulmonary and systemic inflammatory response after hemorrhage in male mice. The authors found that inhalative IL-10 application resulted in a significant decrease of the inflammatory reaction in the lung without affecting the systemic immune response after hemorrhage. From the clinical point of view, generally the lung is the first organ to fail after injury. Furthermore, respiratory failure is most frequent in patients developing MODS, and these patients have the highest mortality rate [4]. Therefore, the results of P. Kobbe et al. have the potential to build the basis for the development of another treatment option after severe trauma.

Priming and activation of leukocytes blood trigger the systemic immune response eventually leading to inflammatory complications. On the other hand, anergy of leukocytes may lead to infectious complications. Neutrophils are the main types of effector cells in the innate immune system [5]. Their activation causes the release of neutrophil extracellular traps (NETs) which exert strong antimicrobial and immunomodulating properties. However, high local NETs concentrations might also result in severe tissue damage with subsequent organ dysfunction. In a study of W. Meng et al. (in this issue) the authors observed the significance of NETs and the natural counter regulator deoxyribonuclease (DNase) for the development of sepsis in multiple trauma patients. They found that levels of NETs and DNase were significantly increased in the very early phase of sepsis or even before clinical manifestation. Therapeutic strategies that limit NETs activity might therefore prevent neutrophil-derived pathological effects possibly resulting in posttraumatic organ failure. Diverse neutrophil CD molecules have been shown to represent reliable marker for the incidence of complications after multiple trauma [2]. However, these parameters have not been implemented in clinical practice due to complex flow cytometric analysis. In this issue M. Groeneveld et al. demonstrated that analysis of CD molecules on a routine haematology analyser is reliable and fast so that these parameters might now be more suitable for implementation in the clinical routine. M. Kolackova et al. (in this issue) found a postoperative increase of systemic IL-10 concentrations which was dependent on the invasiveness of the surgical procedure. Furthermore, they described a correlation between increased IL-10 levels and a higher percentage of neutrophils after surgery. This suggests a functional relationship between both parameters and underlines the complexity of the posttraumatic immune response.

Beside neutrophil function, this special issue focuses on other aspects of the posttraumatic immune response. B. Auner et al. investigated Leukotriene B4, a proinflammatory lipid mediator, as a predictor for pulmonary complications in a population of 100 patients with severe injuries. They demonstrated increased plasma levels of Leukotriene B4 on admission as compared to a healthy control group. Moreover, increased plasma levels of Leukotriene B4 were associated with pulmonary complications but not with the severity of suffered chest injuries. Thus, Leukotriene B4 seems to be a candidate for an early identification of patients on risk for pulmonary complications following major trauma which may have an impact on further therapeutic strategies.

Matrixmetalloproteinases (MMPs) represent another family of inflammatory mediators playing a central role in tissue remodeling of extracellular matrix during the early posttraumatic period. In a clinical study of M. Brumann et al. (in this issue) including 60 polytrauma patients, MMP-9 significantly decreased over a 72 h period while tissue inhibitor of matrixmetalloproteinases- (TIMP-) 1 increased. Furthermore, this effect was dependent on injury severity. Therefore, MMP-9 and TIMP-1 may serve as an indicator for the posttraumatic immune system disbalance.

Proteasomes seem to have a biological role in the extracellular alveolar space, while their role during inflammatory processes remains to be elucidated. In this issue S. U. Sixt et al. found that the total proteasome concentration was increased in the bronchoalveolar lavage (BAL) in patients with ARDS as compared to healthy patients. The immunoproteasome proteins LMP2 and LMP7 were only identified in patients suffering from ARDS. Moreover, the proteasomal enzyme activity pattern was different between patients with ARDS and healthy subjects. Quantitative immunoproteasome measurements in BAL may help to discriminate disease activity and efficacy of therapy.

In the current special issue two new experimental trauma models are introduced. C. Probst et al. presented first results of a murine multiple trauma model including traumatic brain injury, hemorrhage combined with a femoral fracture. The model was characterized by plasma cytokine pattern and lymphocyte subtype populations. In another polytrauma model in rats, the group of M. Huber-Lang et al. combined a closed head injury, chest trauma, and lower leg fracture with soft tissue trauma. The trauma was characterized by systemic and BAL cytokine concentrations and histological analyses as well as changes of the complement system activity.

The current issue provides a broad overview regarding the posttraumatic immune response. Focusing not only on characterization of neutrophils and the impact of cytokines but also on less established parameters like matrixmetalloproteinases and proteasomes, the special issue presents new insights into immunological processes following trauma. The results may serve as an impulse for further research in this field. Molecular mechanisms of the posttraumatic immune response are essential for improvement of treatment strategies in patients suffering from major trauma.

*Frank Hildebrand* *Frank Hildebrand* *Sascha Flohe* *Sascha Flohe* *Loek Leenen* *Loek Leenen* *Martijn van Griensven* *Martijn van Griensven* *Michael Frink* *Michael Frink*
